# Characterization and Extraction Influence Protein Profiling of Edible Bird’s Nest

**DOI:** 10.3390/foods10102248

**Published:** 2021-09-23

**Authors:** Nurul Nadiah Mohamad Nasir, Ramlah Mohamad Ibrahim, Md Zuki Abu Bakar, Rozi Mahmud, Nor Asma Ab Razak

**Affiliations:** 1 Natural Medicine and Products Research Laboratory, Institute of Bioscience, Universiti Putra Malaysia, Serdang 43400, Malaysia; nurul.nadiah.86@gmail.com (N.N.M.N.); ramlah86ibrahim@gmail.com (R.M.I.); zuki@upm.edu.my (M.Z.A.B.); 2 Department of Veterinary Preclinical Sciences, Faculty of Veterinary Medicine, Universiti Putra Malaysia, Serdang 43400, Malaysia; 3 Department of Radiology, Faculty of Medicine and Health Sciences, Universiti Putra Malaysia, Serdang 43400, Malaysia; rozi@upm.edu.my

**Keywords:** food, nutraceutical, edible bird’s nest, *Aerodramus fuciphagus*, half-cup, stripe-shaped, physicochemical, proteomic, protein, extraction

## Abstract

The edible bird nest (EBN) from *Aerodramus fuciphagus* has been consumed as a Chinese traditional food for health and medicinal purposes due to its elevated nutritional value. The present study focused on the influence of characterization and extraction methods on protein profiling, which could be a guideline for grading the EBN. The proposed extraction method is similar to the common food preparation methods of consumers and thus can accurately establish the bioactive protein available upon human consumption. The characterization includes physicochemical analysis (physical, morphology, elemental composition, and microbial content) and chemical analysis (crude protein and amino acid). The morphology of half-cup EBN was found to be uniformly shaped and rich in calcium as compared to rough surface of stripe-shaped EBN, and there was no significant microbial growth in both types of EBN. The crude protein and amino acid content in half-cup EBN were significantly higher than stripe-shaped EBN. The full stew (FS) and stew (SE) extraction methods produced a maximal yield of soluble protein. Sialic acid content in SE extract (8.47%, *w/w*) and FS extract (7.91%, *w/w*) were recorded. About seven parent proteins (39.15 to 181.68 kDa) were identified by LC-MS/MS Q-TOF, namely 78 kDa glucose-regulated protein, lysyl oxidase-3, Mucin-5AC-like, acidic mammalian chitinase-like, 45 kDa calcium-binding protein, nucleobindin-2, and ovoinhibitor-like. In conclusion, the characteristics and extraction methods influence the availability of bioactive protein and peptides, demonstrating the potential usage of EBN in improving its biological activities and nutritional properties.

## 1. Introduction

The edible bird’s nest (EBN) is the nest of the swift. It is constructed with the saliva secreted from the pair of its sublingual glands as the main material. *Aerodramus fuciphagus* (white nests) and *Aerodramus maximus* (black nests) are the two main species of swiftlets that are known to produce valuable EBNs. Malaysia is the second largest exporter in the world, contributing about 20% to the total market of EBN production. Less than half of the EBN produced are consumed by locals, while a larger percentage is exported to other countries, such as Hong Kong, Singapore, and China [1]. Due to its high nutritional and medicinal therapeutic values, EBNs can cost USD 2000–10,000 per kilogram and are regarded as the most expensive animal by-product in the world [2]. White EBNs are regarded as the “Caviar of the East” due to their unique taste and smooth texture, they have also been widely used by people, especially the Chinese community, as traditional medicines. In traditional Chinese medicine, EBNs are prescribed to treat diseases such as tuberculosis, asthma, dry cough, hemoptysis, asthenia, difficulty in breathing, gastric troubles, and general bronchial ailments [3].

Interestingly, EBNs have also been the focus of modern research and technology, which have revealed their nutritional value and pharmacological activities, including anti-aging, anti-cancer, cough-suppressing, anti-tuberculosis, voice-improving, and phlegm-dissolving properties [4]. Meanwhile, in the food industry, EBN extract has been used as one of the ingredients in foods, drinks, and nutraceutical products. For example, ice cream with 0.2% EBN extract was found to be more favorable than the others due to high melting rates and lower sweetness [4]. These positive nutritional and health effects have increased the demand and supply of EBNs and have generated increasing interest among researchers. Investigations of the hidden nutritive and pharmacological properties of EBNs have become the primary focus of this research due to their potential as a therapeutic agent.

EBNs consist of protein (42.0–63.0%), carbohydrate (10.63–27.26%), moisture (7.5–12.9%), ash (2.1–7.3%), and fat (0.14–1.28%) [5]. Bioactive peptides released from EBN hydrolysate can exhibit various biological activities and nutritional properties, such as lowering blood pressure by ACE inhibitory peptides [6]. The main carbohydrate found in EBNs is sialic acid, which is a type of glycoprotein that consists of oligosaccharide chains (glycans) covalently attached to amino acid sidechains. Sialic acid has been proven to ameliorate cardiovascular disease biomarkers [7] and significantly improve the learning and memory function of the preterm infant [8]. To date, EBN containing bioactive peptides were found to improve bone loss and skin aging in post-menopausal women [9], to be effective in the treatment of neurodegenerative diseases [10], to prevent obesity-related inflammation and oxidative stress in rats [11], to lower oxidative stress and inflammatory markers [12], and to improve spatial learning performance in children [13].

Consumption of EBN-based nutritional beverages has been found to promote the improvement of human health. EBN glycopeptides were used in the formulation of ready-to-drink products and have been shown to have significantly higher antioxidant activity (*p* < 0.05) compared to non-EBN drinks [14]. In the cosmetic industry, EBN extracts have been incorporated into skincare products and have been used to promote skin cell renewal, radiant complexion, and anti-aging [4]. In some studies, EBN has been shown to improve digestive problems [5], with the prebiotic properties of EBN promoting a healthy human gut environment.

The bird’s nest industry in Malaysia suffered a setback in 2011 when China banned EBN exports due to high concentrations of nitrate, lead, and arsenic detected in certain EBN products. As a result, strict regulations regarding EBN standards and specifications were implemented and enforced. The requirement listed in the EBN protocol for China specifies that EBN products must be free from avian influenza and the nitrite content must be less than 30 ppm. The quality of EBNs from Malaysia for export to China is set by [15], a Malaysian Standard that includes (1) MS 2333:2010 Good manufacturing practice (GMP) for processing raw-unclean and raw-clean edible birdnest (EBN); (2) MS 2334:2011 Edible birdnest (EBN)-specification; (3) MS 2612:2015 Raw-unclean edible birdnest (EBN)-house nest specification; and (4) MS 2509:2012 (P) Test method for edible bird nest (EBN)-determination for nitrite (NO_2_^−^) and nitrate (NO_3_^−^) content. The Malaysian Standards also provide requirements for EBN swiftlet farming—MS 2273:2012 and MS 2333:2010—as references for the EBN industry to reduce potential contamination in EBNs. The Standard MS 2273:2012 specifies guidelines for ranching practices of edible nest swiftlets, including ranch design and maintenance, hygiene of the premises, and signs of illness in swiftlets. The Standard MS 2333:2010 provides guidelines for the design of processing facilities in order to avoid cross-contamination and to control procedures that affect operations, building maintenance, personal hygiene, and animal hygiene control systems (contaminant control).

Grading of harvested EBNs depend on factors such as shape (half-cup or stripe-shaped), type (white, red, or grass), color (white, yellow, or red), and cleanliness of the nest. In addition, the dry mass, duration of nest building, and protein content of cleaned house-farmed EBNs also contribute to the EBN grading, reflecting quality [5]. The detailed characteristics and grading of EBNs, including physicochemical analysis (physical [12], morphology [16], elemental composition [17], and microbial content [18]) and chemical analysis (crude protein [2], amino acid [19] and sialic acid [7]) have been reported. However, research on the relationship between the physical and chemical characteristics of EBNs and their nutritional and bioactive properties is lacking.

Several methods have been reported for obtaining EBN extract and bioactive peptides, such as solvent extraction, heat extraction, enzymatic hydrolysis, and microbial fermentation [4]. Solvent extraction may result in low extraction efficiency, low selectivity, solvent residue, and environmental pollution. In contrast, enzymatic hydrolysis is preferable compared to solvent extraction and microbial fermentation due to high recovery, less solvent residue, and being more environmentally friendly, making this method popular among food and pharmaceutical industries [20]. However, data on the impact of characterization and extraction of different shapes of EBNs on the protein profiling and bioactive content is insufficient; this research area needs to be explored for a better understanding of EBN applications. A bioactive compound, when extracted using different extraction methods, may potentially produce a wide range of bioactivity, which may be attributed to the synergistic effects of diverse constituents of the food [21].

Proteomic research into EBNs has resulted in a bottleneck, due to its poor solubility and low extractive rate [22] and the limited number of protein sequences deposited in the database. The effects of simulated gastrointestinal digestion provide insight into the amounts of bioactive peptides that are likely to be derived from hydrolysis of EBNs in real conditions of humans. The optimum conditions implicated for the bioavailability of EBN glycopeptides is thus double boiling followed by enzymatic hydrolysis; this combination of methods uncoils the glycoprotein structure, which in turn may unlock the potential benefit of EBNs [4].

Even though both half-cup and stripe-shape EBNs originate from the same source, the comparison data from physicochemical analysis (physical, morphology, elemental composition, and microbial content) and chemical analysis (crude protein, amino acid, and sialic acid) is important to understand and correlate between protein hydrolysis and bioactive peptide studies. Four types of water extraction combined with heat treatment were selected to simulate the common traditional preparation of EBN, aimed to profile the water-soluble protein. Protein profiling portrayed by SDS-PAGE and LC-MS/MS analysis demonstrates confirmation of the good quality of protein produced by the hydrolysis technique, which is related to the functional characterization of bioactive glycopeptides. This peptide sequence clarified the specific bioactive activity with different characteristics, which is important for food, pharmaceutical, and medical applications.

## 2. Materials and Methods

### 2.1. Edible Bird’s Nest (EBN)

Different types (half-cup and stripe-shaped) of raw-cleaned house Edible Bird’s Nest (EBN) were purchased from Blossom View Sdn. Bhd in Terengganu (East Coast of Malaysia). Feathers and impurities identified during the screening procedure were manually removed using tweezers, ground with a mortar to form EBN powder, and stored in an air-tight container at 4 °C until further use.

### 2.2. Characterization

#### 2.2.1. Physicochemical Analysis (Physical, Morphology, Elemental Composition, and Microbial Content)

EBNs with half-cup and striped-shaped physical characteristics were measured by a ruler in triplicate. The morphology characteristics were obtained by fixing EBN powder to a stub with carbon tape, coating with gold, and placing on the sample holder. Next, the sample holder was fixed on a rotatable disc inside the machine and observed using a scanning electron microscope (SEM) (FEI Quanta 400F). Meanwhile, energy dispersive X-ray spectroscopy (EDX) was used to investigate the elemental constituents; the atomic and weight percentage of the elements were determined under low vacuum at an accelerating voltage of 15 kV and a current of 60–90 mA. The microbiological analysis for both EBN types was performed according to standard methods specified by the Food and Drug Administration/Bacteriological Analytical Manual (FDA/BAM) and Official Methods of Analysis of Association of Analytical Chemists (AOAC), 16th Edition (1995). The FDA/BAM analytical methods were used to identify aerobic plate count (APC) (Chapter 3), yeasts and molds (Chapter 18), *Staphylococcus aureus* (*S. aureus*) (Chapter 12), and *Salmonella* (Chapter 5) [23], while AOAC: 991.14 (1995) was used to identify coliforms and *Escherichia coli* (*E. coli*) in the EBNs [24].

The APC was used to indicate the level of microorganisms in products. Briefly, the EBN sample (10 mL) was mixed with diluent (90 mL) to obtain 10^−2^, 10^−3^, and 10^−4^ decimal dilutions. The inoculated diluents sample (1.0 mL) was mixed with plate count agar in a Petri dish and incubated aerobically at 37 °C for 48 h. Meanwhile, the yeast and mold count in the EBN samples were determined out using a media supplemented by 100 mg/L chloramphenicol to inhibit bacterial growth. Peptone water (0.1%) was added to the EBN sample (25–50 g) and homogenized for 2 min. The solution (0.1 mL) was pipetted onto solidified dichloran rose bengal chloramphenicol (DRBC) agar and subjected to incubation in a dark place at 25 °C for 5 days. Coliforms and *E. coli* in EBN samples were determined using 3 M Petri film (3M Co., Saint Paul, MN, USA). The film was placed on a flat surface and inoculated with 1 mL of test suspension onto the center of the film base. The plate was incubated at 37 °C for 24 h. The red and blue colonies referred to coliforms and *E. coli,* respectively.

The presence of *S. aureus* in EBNs was examined by transferring 1 mL of EBN sample onto a Baird-Parker agar plate. The inoculum was spread evenly using a sterile bent glass streaking rod, and the plate was then incubated at 35–37 °C for 45–48 h. Detection of *Salmonella* in EBN was carried out by mixing a 25 g sample and 225 mL sterile lactose broth in a sterile blending container. The mixture was transferred into a sterile, wide-mouth, screw-cap jar and left at room temperature for 60 ± 5 min, with the jar securely capped. Then, the mixture was mixed well, and the jar cap was slightly loosened before incubation at 35 °C for 24 ± 2 h. Rappaport-Vassiliadis (RV) medium (1:100) and tetrathionate (TT) broth (1:10) were added, and the mixture was subsequently incubated as follows: RV: 24 ± 2 h at 42 °C; TT: 24 ± 2 h at 35 °C. About 10 μL of incubated TT broth was then used to streak 3 mm loopful on bismuth sulfite (BS) agar, xylose lysine deoxycholate (XLD) agar, and Hektoen enteric (HE) agar. The loopful step was repeated using RV medium (10 μL) on the same agar plates before being incubated for 24 ± 2 h at 35 °C. The results for APC, yeasts and molds, coliforms, and E. coli were expressed as log_10_ colony-forming unit per gram (CFU/g) EBN. Meanwhile, the result for *S. aureus* was reported as the most probable number per gram (MPN/g) of EBN, and *Salmonella* species were reported as either detected or not detected in 25 g of EBN.

#### 2.2.2. Chemical Analysis (Crude Protein and Amino Acid)

The crude protein in both EBNs was determined by the Kjeldahl method, using 6.25 as a conversion factor [25]. About 500 mg of EBN, 1 tablet of a catalytic amount of CuSO_4_/K_2_SO_4_, and 12 mL of sulfuric acid were added into the digestion tube to initiate the digestion process, until clear green or blue solution was obtained. The solution was cooled for 10–20 min before the addition of 75 mL of distilled water. The analysis was continued with the distillation process through the addition of 25 mL boric acid and 10 drops of bromocresol green as an indicator. Next, the cooled digestion tubes were placed in the digestion unit, and 50 mL of sodium hydroxide solution was added to samples for 5 min in a distillation unit. The distillate was titrated with hydrochloric acid until the changed color was obtained and was calculated in accordance with [26].

The amino acid composition was analyzed based on [27] using high-performance liquid chromatography (HPLC). About 0.3 g of EBN sample was hydrolyzed with 5 mL of 6 N HCl at 110 °C for 24 h. The samples were cooled to room temperature and filtered through filter paper into a 100 mL volumetric flask. The 400 μL of internal standard (50 μmol/mL α-aminobutyric acid (AABA) in 0.1 M HCl) was added into the same flask and topped up to 100 mL with distilled water. The aliquot was filtered by using a 0.20 mm polytetrafluoroethylene microfilter. As for derivatization, 10 μL filtered hydrolysate samples or standard and 70 μL borate buffer solution were mixed well into a 1.5 mL glass vial. Then, 20 μL AccQ Flour reagent (3 mg/mL in acetonitrile) was added to the mixture and vortexed for a few seconds. The 10 μL of samples and standards was injected into the HPLC (Waters 2475, Waters Co., Milford, MA, USA), with a 1 mL/min flow rate. Amino acid analysis was performed using AccQ Tag (3.9 × 150 mm) column with mobile phase A (Eluent A—200 mL AccQ Tag to 2 L of Milli-Q water) and mobile phase B (Eluent B—60% acetonitrile). The linear gradient condition was set according to the following times: 100% A and 0% B (start), 98% A and 2% B (0.5 min), 91% A and 9% B (15 min), 87% A and 13% B (19 min), 65% A and 35% B (32 min), 65% A and 35% B (34 min), 0% A and 100% B (35 min), 0% A and 100% B (38 min), 100% A and 0% B (39 min) and 100% A and 0% B (50 min). Amino acid content was read at 250 nm using a fluorescence detector (λ excitation and λ emission).

### 2.3. EBN Extraction

EBN extraction was performed according to a previous study [28], with some modifications. Water extraction was selected to simulate the traditional technique commonly used before the consumption of EBNs. Water extraction gives a general idea of the protein consumed by people. EBN extraction was done using four types of methods: stew (SE), full stew (FS), sonication (SO), and hot water (HW). The cleaned EBN was ground and soaked with deionized water, with a ratio of 1:100 (*w/v*), for 16 h at 4 °C. All the extracts were subsequently frozen at −80 °C, lyophilized, and stored at −20 °C until further use.

#### 2.3.1. Stew and Full Stew Extraction

SE and FS extraction methods were carried out using a double-boiling technique, where the vapor from the boiling water in the bottom pot heated the top pot containing the EBN. The soaked EBN was double-boiled for 30 min at 100 °C and cooled to room temperature. The SE extract consisted of the double-boiled EBN, filtered using a muslin cloth, and the FS extract consisted of the whole double-boiled EBN, without filtration.

#### 2.3.2. Sonication

SO extraction was performed by sonicating soaked EBN in a beaker for 30 min at room temperature (25 ± 1 °C) using an ultrasonic cleaner (42 kHz, 135 W; Branson Ultrasonic Corporation, Fairfield County, CT, USA). The water level in the beaker was kept at the same level of water as the ultrasonic bath, maintained at a constant room temperature (25 ± 1 °C). Next, the aliquot was filtered using a muslin cloth to obtain the extract.

#### 2.3.3. Hot Water

Direct heat from boiling water was used to obtained the HW extract. The soaked EBN was placed in a beaker and heated for 30 min at 100 °C in a water bath. It was then cooled to room temperature and subsequently filtered through a muslin cloth to obtain the extract.

### 2.4. Determination of Sialic Acid by HPLC

The sialic acid content was evaluated based on [13] using high-performance liquid chromatography (HPLC) (Agilent1200) with a reversed-phase Agilent HC-C18 column (4.5 × 250 mm, 5 μm). The methanol, acetonitrile, and water solution (7:8:85) were used as a mobile phase, with a flow rate of 0.9 mL/min. A fluorescent detector was used, with the excitation wavelength at 373 nm and emission wavelength at 448 nm.

### 2.5. Enzymatic Hydrolysis of EBN

Enzymatic hydrolysis, following simulated gastrointestinal digestion, was performed to produce edible bird’s nest (EBN) protein hydrolysate as previously described by [3], with slight modifications. The pH of EBN extract (5 mg/mL) was adjusted to 2.0 using HCl (20 mL) and digested with pepsin (1% (*w/w*) in 0.1 M potassium chloride). The pH-adjusted sample was put in a shaking incubator for 2 h at 37 °C and boiled for 20 min to stop the pepsin activity. The pH of the mixture was increased to 8 with 1 M NaOH and further digested with pancreatin (1% (*w/w*) in 0.1 M potassium phosphate buffer) for another 2 h at 37 °C to simulate small intestine conditions. The reaction was stopped by boiling the sample for 30 min and immediately cooling at pH 8.9. The hydrolyzed solution was centrifuged at 10,000× *g* at 4 °C for 30 min and desalted using snakeskin pleated dialysis tubing at 7.0 K MWCO (Thermo Fisher Scientific Inc., Watham, MA, USA). The hydrolysate was subsequently frozen at −80 °C, lyophilized, and stored at −20 °C until further use.

### 2.6. Estimation of Soluble Protein

The soluble protein content of EBN samples was determined using the Bradford protein assay kit following the manufacturer’s instructions (Catalog Number: P010; Gene Copeia, Rockville, MD, USA) [29]. About 100 μL of the sample and bovine serum albumin (BSA) standard were added into 96-well plate, followed by the addition of 100 μL of Bradford reagent to each well. The mixture was mixed with a plate shaker for 30 s and incubated for 5 min at room temperature. Protein concentration was calculated according to the standard protein curve of BSA, and absorbance was read at 595 nm.

### 2.7. Protein Separation and Molecular Weight Determination Using SDS-PAGE

The samples for purification and determining the molecular weight of the protein were prepared using optimized half-cup EBN extracts (FS and SE) and hydrolysates (FSh and SEh). The sodium dodecyl sulfate-polyacrylamide electrophoresis (SDS-PAGE) was prepared according to [30], with some modifications. The 5% (*w/v*) of EBN was loaded in 12% resolving gel and 4% stacking gel in a 1:1 (*v/v*) ratio containing Tris buffer. The solution was heated in a 90 °C water bath for 20 min and then cooled immediately. Then, 20 μL of the sample and 20 μL of the protein standard were loaded into individual wells and run under the constant current setting of 30 mA and 150 V for 15 min before being increased to 200 V for another 45 min. Proteins were stained with 0.1% (*w/v*) Coomassie blue, with protein markers in the range of 11 to 245 kDa.

### 2.8. Protein Identification by LC-MS/MS Q-TOF

#### 2.8.1. EBN Protein Digestion

In-solution digestion of the EBN sample was carried out according to the manufacturer’s instructions [31]. RapiGest solution (0.2%, *w/v*) was prepared by resuspending 1 mg RapiGest^TM^ (Waters) in 500 μL of 50 mM ammonium bicarbonate. EBN extract (100 μg) was then dissolved in 50 μL of 0.2% RapiGest solution and vortexed. DTT was added to the mixture to a final concentration of 5 mM for the reduction step before boiling at 60 °C for 30 min. The mixture was cooled to room temperature, before being alkylated with iodoacetamide to a final concentration of 15 mM for 30 min in the dark environment. The proteolytic digestion step was performed by adding mass spectrometry grade Trypsin Gold (Promega, Madison, WI, USA) at a ratio of 1:50 (trypsin/protein), followed by incubation overnight at 37 °C. At the end of the digestion step, 1 μL of formic acid was added to stop trypsin activity. The digested protein samples were stored at −20 °C before protein and peptide identification.

#### 2.8.2. Liquid Chromatography-Tandem Mass Spectrometry Coupled Quadrupole-Time of Flight (LC-MS/MS Q-TOF) Analysis

LC-MS/MS analysis was performed using 6550 iFunnel Q-TOF LC/MS from Agilent Technologies (Santa Clara, CA, USA). A total of 5 μL digested EBN was loaded into an Agilent Large Capacity Chip consisting of a 75 μm × 150 mm analytical column and a 160 mL enrichment column, which was packed by 5 mM of Zorbax 300SB-C18 for chromatographic separation. The mobile phase consisted of solvent A (0.1% formic acid in MilliQ water) and Solvent B (9:1 ratio of 0.1% formic acid in acetonitrile: MilliQ water); a flow rate of 1.0 mL/min was used to elute the peptides. The mobile phase gradient was programmed as 3–50% of solvent B for 30 min; 50–95% of solvent B for 2 min; 95% of solvent B for 7 min; and 95–3% of solvent B for 47 min. The polarity of Q-TOF was set at positive, the voltages for capillary (2050 V) and fragment (300 V) were set accordingly, and the gas flow was set at 5 L/min and 325 °C. The peptide spectra were acquired using Agilent MassHunter Workstation Data Acquisition software (Agilent Technologies, Santa Clara, CA, USA) by monitored positive ion acquisition in the range of 110 to 3000 m/z for the MS scan, and 50 to 3000 m/z for the MS/MS scan. The chromatograms obtained were analyzed using the Agilent MassHunter Qualitative Analysis B.05.00 software (Agilent Technologies, Santa Clara, CA, USA).

#### 2.8.3. Data Analysis

A Swiss-Prot database search for protein and peptide identification matched with *Charadrius vociferus* species was performed using the Spectrum Mill MS Proteomics Workbench (Agilent Technologies, Santa Clara, CA, USA). Carbamidomethylation was selected as the fixed modification parameter and trypsin as the digestive enzyme, with the maximum number of cleavage equal to 2. Auto-validation was set for MS searches at a false discovery rate (FDR) of 1.2% and data export to Mass Profiler Professional (MPP v 14.9.1, Agilent Technologies, Santa Clara, CA, USA) for further analysis. The software confirmed the identified protein and the post-translation modifications if present, and the data files were then processed by principal component analysis (PCA) using the MPP software.

### 2.9. Statistical Analysis

Statistical analysis was performed using SPSS version 24.0. All results were expressed as mean ± standard deviation (SD). The data were statistically treated by paired-sample t-test and analysis of variance (ANOVA), with *p* < 0.05 considered to be statistically significant, and the mean was compared using Duncan’s multiple range tests.

## 3. Results and Discussion

### 3.1. Physicochemical Analysis (Physical, Morphology, Elemental Composition, and Microbial Content)

**Figure 1:**
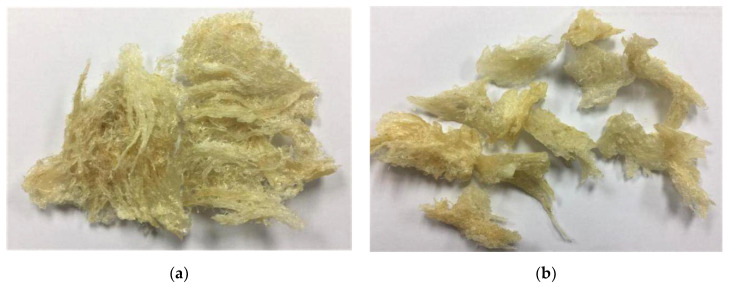
EBN image: (**a**) half-cup and (**b**) stripe-shaped.

The physicochemical analysis included physical measurement, chemical composition, and microbial content of half-cup and stripe-shaped EBNs. The nest cups used were half-cup and stripe-shaped, as shown in Figure 1. The half-cup EBN is U-shaped, and the stripe-shaped EBN is a hard, incomplete cup shape, with some broken parts. Table 1 shows the physical measurement of half-cup and stripe-shaped EBNs. The height, length, and weight were 4.08 ± 0.71 cm, 7.06 ± 1.33 cm, and 5.19 ± 0.18 g (half-cup) and 1.28 ± 0.15 cm, 3.26 ± 0.48 cm, and 2.26 ± 0.18 g (stripe-shaped), respectively; the weight of stripe-shaped EBN was half of that of the half-cup EBN, and the height, length, and weight between half-cup and stripe-shaped EBNs were statistically significantly different (*p* < 0.05). Similar findings of average height, length, and weight of EBNs have also been reported [16], ranging from 3.5 to 5.0 cm, 7.0 to 13.0 cm, and 5.6 ± 1.3 g, respectively. The shape of an EBN is crucial for grading purposes, whereby the price is determined. The shape of the half-cup EBN is Grade A, and the stripe-shaped EBN derived from the broken part of the half-cup EBN is classified as Grade B.

**Figure 2:**
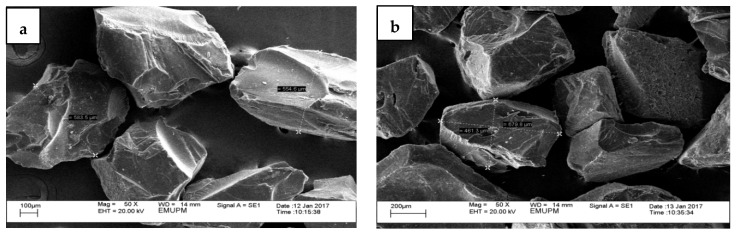

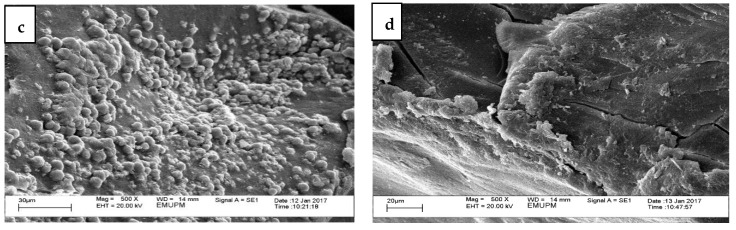
Scanning electron micrograph of EBNs at magnification of 50× for (**a**) half-cup and (**b**) stripe-shaped, and magnification of 500× *g* for (**c**) half-cup and (**d**) stripe-shaped.

Figure 2 shows the surface morphology of half-cup and stripe-shaped EBNs at 50× and 500× magnification by SEM. These structures are seen to be coated with a layer of a partially clear or transparent substance. Substances appearing rough and hard covered some of the surfaces of the EBNs. Micrograph images at 500× magnification showed that the EBN was unevenly structured. The surface formation of the half-cup EBN was uniformly shaped, while a rough surface can be seen in the stripe-shaped EBN. Visualization under 50× magnification for both half-cup and stripe-shaped EBNs revealed ground EBN as an irregular prism with a crystalline structure. Irregular prisms with lustrous translucent to opaque silver crystalline structures were also reported by [16]. The washing and drying process is a common step in processing EBNs, to increase the shelf-life, lower the water activity, and facilitate storage before manufacturing and packaging. Thus, no foreign or unwanted particles, such as mites, fungal structures, and feather strands, were observed. However, drying involving heat can cause a crystalline appearance, which was observed in both half-cup and stripe-shaped EBNs using SEM.

SEM with energy-dispersive X-ray (EDX) for elemental analysis was done to characterize the composition and chemical characteristics of both EBNs, as shown in Table 1. Carbon and oxygen elements in stripe-shaped EBNs were five-fold higher than half-cup EBNs. Magnesium (0.22%) was only found in the stripe-shaped EBN sample. Oxalic acid secretion, calcium availability, hydration state, and other environmental factors may influence crystal production and morphology [17]. The high calcium content in the half-cup EBN can be assumed from the translucent crystalline structure (Figure 2a), which is glassier than the stripe-shaped EBN (Figure 2b) under the same resolution at 50×; this structure is supported by calcium, which in the half-cup EBN is two-fold higher (72.94%) than calcium element in striped-shaped EBN (44.16%).

Microorganisms such as coliforms, *Escherichia coli* (*E. coli*), *Salmonella*, *Staphylococcus aureus* (*S*. *aureus*), yeast, and mold were also quantified (Table 1). The results showed that both EBNs had a similar number of aerobic plate counts (1.8 × 10^8^ CFU/g), coliforms (1.4 × 10^4^ CFU/g), *E. Coli* (not detected at less than 10 CFU/g), *Salmonella* (absent), and *S. aureus* (not detected at less than 3 CFU/g). However, 67 CFU/g of yeast and mold were detected in the half-cup compared to stripe-shaped EBN. Bacteria present in EBNs may lead to food-borne disease if ingested by a human. *Salmonella sp.* and *E. coli* are the most common food-borne pathogens that can affect health conditions; they can cause severe diarrhea or meningitis, which can be fatal if untreated [32]. Based on the latest standards set by the Ministry of Health Malaysia, both types of EBN used in this study were in accordance with the specified standard that raw-clean EBNs should be free from *E. coli*, *Salmonella* sp., and *S.* *aureus* before being exported to another country [18]. The Standard and Industrial Research Institute of Malaysia (SIRIM) provides guidelines for Edible Bird Nest (EBN) (MS 2334:2010), including the permitted levels of microbial content: total plate count (<2.5 × 106 CFU/g), coliform (<1100 most probably number (MPN)/g), *E. coli* (<100 (MPN)/g), *S. aureus* (<100 (MPN)/g), yeast and mold (<10 CFU), and no presence of *Salmonella sp.* [33].

Yeast, mold, and other contaminants such as mites and feathers can be located within the strands of EBNs. This was shown in the quantification analysis, after the EBN was ground into small pieces. Thus, it is important to educate consumers on the need for additional washing and boiling, which can assist in removing contaminants [33] and significantly reduce the number of bacteria before consumption [18].

### 3.2. Chemical Analysis (Crude Protein and Amino Acid)

Table 2 shows the crude protein content and amino acid profile of half-cup and stripe-shaped EBNs. A higher crude protein content in the half-cup (56.96 ± 0.09%) than stripe-shaped (54.70 ± 0.16%) EBN was obtained, which was significantly different for both EBNs (*p* < 0.05). These results were comparable with EBN samples from East Coast Peninsular Malaysia, ECM (Pahang: 55.48 ± 3.60%) [2], Northern Peninsular Malaysia, NM (Perak, Penang, Kedah: 53.8 ± 0.18%) and East Malaysia, EM (Sabah, Sarawak: 52.8 ± 1.04%) [18]. The major amino acids (AAs) found in both half-cup and stripe-shaped EBNs were glutamic acid (Glu), followed by aspartic acid (Asp), serine (Ser), and valine (V), which were also reported in EBN samples from ECM [2]. The similarity in the protein and amino acid profiles of the EBN samples studied by [2] and those of present study was due to the EBN samples being collected from the same region (ECM). However, the major amino acids in EBN from EM [18] were different due to different collection locations (NM and EM). Overall, both half-cup and stripe-shaped EBNs possess a high percentage of amino acids and nonessential amino acid composition. These variances could be due to different locations of EBN harvesting, the effects of processing methods, etc. Breeding sites, climate, and swiftlet diet may also affect the EBN nutrient composition [18].

The nutritional value of essential amino acids (EAAs) composition contained in protein may contribute to the functional biopeptides. The readily digestible dietary EAAs are needed at an optimum level for human body requirements, e.g., EAA Gly supplementation has been reported to promote anti-inflammatory effects during endothelial inflammation, while Arg has been found to improve endothelial function in cardiovascular or overweight patients [34]. The presence of nEAA Glu in the body is very important, especially for transamination reactions in amino acid metabolism. It is involved in the synthesis of key molecules such as glutathione, which is important in the alleviation of oxidative stress and modulation of the immune response [35]. Asp plays a role as a regulator in hormone secretion and acts as a precursor for methionine (Met), threonine (Thr), isoleucine (Ile), and lysine (Lys). Similarly, Ser is the precursor of glycine (Gly), cysteine (Cys), and tryptophan (Trp) and is involved in cell signaling.

### 3.3. EBN Extracts and Soluble Protein Concentration

**Figure 3:**
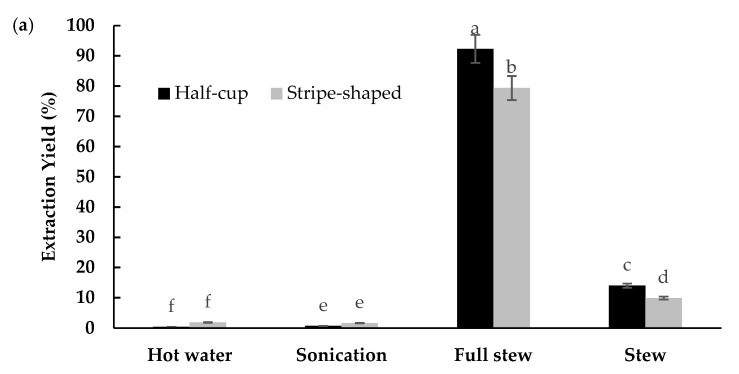

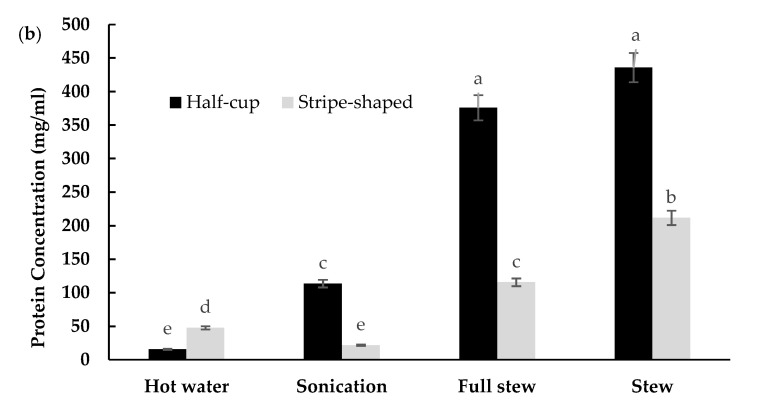
The (**a**) extraction yield and (**b**) protein concentration of half-cup and stripe-shaped EBNs using different extraction methods. Data shown are means ± standard deviation of triplicates. a,b,c,d,e,f: means with different letters indicate significant differences among the extraction methods and types of EBN (*p* < 0.05).

EBN extraction is affected by its solubility behavior towards water. Since protein is the major compound in EBNs, preliminary experiments found temperature affected the extracted protein of the EBN. The extraction yield of half-cup and stripe-shaped EBNs is shown in Figure 3a. The FS method showed the highest extraction yield (half-cup = 92.29 ± 2.45%; stripe-shaped = 79.35 ± 0.91%) as compared to the SE method (half-cup = 12.50 ± 0.89%; stripe-shaped = 10.99 ± 0.11%), with significant difference (*p* < 0.05). Meanwhile, less than 5% extraction yield was obtained using the SO and HW methods for both EBNs. The temperature recorded for the double-boiling technique applied for FS and SE was 73 °C to 80 °C; SO, 25 °C; and HW, 100 °C.

The double-boiled technique with indirect heating contributes to the maximal yield of the FS and SE extraction methods. There was a significant difference in extraction yield between SE and FS methods for both EBNs (*p* < 0.05), while no significant difference was observed in both EBNs using the SO and HW methods (*p* > 0.05). Thus, the different temperatures and the characteristics of the different EBNs affected the extraction yield, with the optimum temperature between 73 °C and 80 °C. Meanwhile, low temperature (below 60 °C) or high temperature (more than 80 °C) resulted in a low extraction yield, as seen with the SO and HW extraction methods, respectively. These results agree with findings reported by [16], emphasizing that the protein concentration of the EBN extract drastically increases under extraction temperatures between 60 °C and 80 °C, but gradually reduces above 80 °C. Thus, temperature influences the extraction and solubility of proteins in EBNs.

Figure 3b shows the soluble protein concentration of EBN extracts obtained from four different extraction methods. The highest protein concentration was in the half-cup EBN (SE = 435.6 mg/mL; FS = 375.6 mg/mL; SO = 113.6 mg/mL) compared to the stripe-shaped EBN (SE = 211.6 mg/mL; FS = 115.6 mg/mL; SO = 21.6 mg/mL), while the lowest soluble protein concentration was obtained in the HW extract (half-cup = 15.6 mg/mL). There were significant differences in soluble protein concentration between the two EBNs for the extraction methods applied (*p* < 0.05), but protein concentration of the half-cup FS and SE extracts were not significantly different (*p* > 0.05).

The extraction yield and soluble protein concentration in the SE method were inversely proportional with temperature. The high yield of the FS extract was a combination of soluble and insoluble fractions of the half-cup EBN, while the SE extract was mainly in soluble form. According to [36], water extraction produced the highest protein content as compared to other extraction methods, indicating that EBNs have an abundance of water-soluble proteins. Thus, the concentrated soluble protein fraction in the SE extract was contributed by its protein concentration. The protein in the insoluble fraction of the FS extract may not fully break down during the double-boiling process, resulting in the incomplete release of soluble protein. The high temperature of the HW methods (100 °C) may cause denaturation of the protein, and the mild heat of the SO methods (25 ± 1 °C) produced an incomplete breakdown of complex protein, resulting in low yield and low soluble protein concentration in both extraction methods.

The optimum extraction condition needs to be chosen to obtain the highest protein concentration and bioactivity of EBNs for further application. The FS and SE extraction methods produced a high yield of crude extract and protein concentration in the half-cup EBN compared to the stripe-shaped EBN. Thus, FS and SE extraction methods for the half-cup EBN were selected to be used for sialic determination and enzymatic hydrolysis.

### 3.4. Sialic Acid Content of EBN Extracts

The SE extract of the EBNs contains higher sialic acid than the FS extract, with 8.47% (*w/w*) and 7.91% (*w/w*), respectively, compared to other findings of 8.6% [4] and 11% [11]. In addition, the sialic acid content in EBNs from Malaysia are varied, with 0.70% to 1.50% from North, South, and East Peninsular Malaysia [37] and 1.17% to 3.15%, collected from North, South, and Borneo Sabah [8]. The types of habitats, environment surroundings, breeding site, nest harvesting season, and diet of the swiftlets are the factors that influence the sialic acid content of EBNs from different locations [8].

These findings agree with the protein content of half-cup EBNs in the present study, suggesting that high protein may contributed to high sialic acid content in EBNs. This is because sialic acid is a component of the glycoprotein located in the carbohydrate chains attached to soluble proteins. This results in a high sialic acid content in the SE extract as compared to FS extracts. As mentioned earlier, the soluble and an insoluble fractions of the FS extract resulted from the incomplete breakdown of protein and may reduce the water-soluble bound sialic acid contents.

Both proteins and sialic acid are major nutraceutical ingredients in EBNs. In a study conducted using EBN constituents producing synergistic antioxidative effects, sialic acid was found to ameliorate the progression of atherosclerosis and other cardiovascular disease biomarkers [7]. Sialic acid in EBNs has also shown potential antiviral properties by inhibiting viral genes, strengthening the lungs, improving skin health, and showing anti-aging properties [4]. Taken together, the findings of crude protein, amino acids, and sialic acid analyzed in the half-cup and stripe-shaped EBNs show that EBNs are a good source of protein and glycoprotein, potentially exhibiting nutritional and medicinal properties.

### 3.5. Soluble Protein Concentration of EBN Hydrolysates

**Figure 4:**
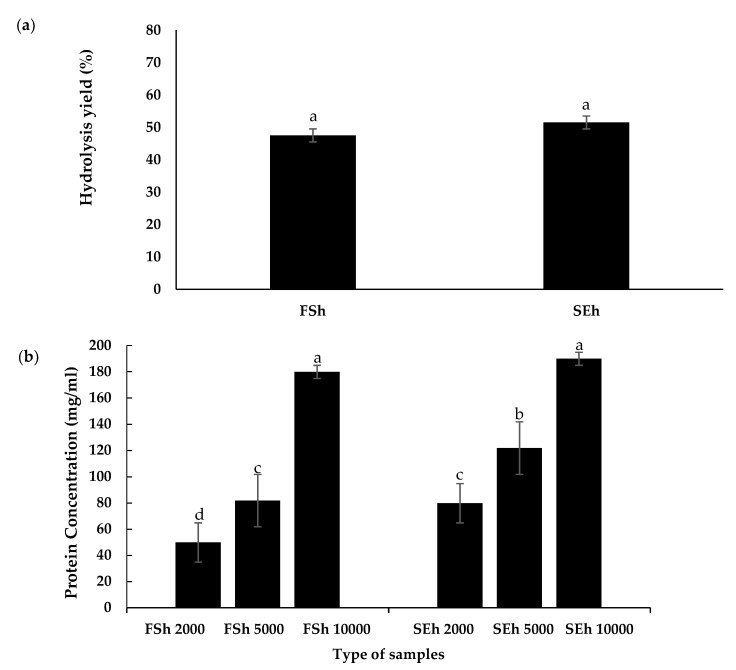
The (**a**) hydrolysis yield and (**b**) protein concentration of half-cup EBN hydrolysate. Data shown are means ± standard deviation of triplicates. FSh: full stew hydrolysate; SEh: stew hydrolysate. ^a,b,c^ Different letters indicate a significant difference between FS and SE hydrolysates at different concentrations (*p* < 0.05).

The high soluble protein found in the half-cup EBN as reported in Section 3.3 indicates a protein-rich sample that yielded a high amount of hydrolysate. The yield of the half-cup FSh (47.5%) was comparable to half-cup SEh (51.5%), as shown in Figure 4a. Enzymatic hydrolysis potentially breaks down complex protein and helps in releasing bioactive peptides from the inactive parent protein, which enables them to exhibit biological properties for therapeutic purposes [6]. Figure 4b shows an uptrend pattern of soluble protein concentration in EBN hydrolysates. Solubility is primarily dependent on the distribution of hydrophobic and hydrophilic amino acids on the surface of the protein and is affected by the protein–water interaction.

A similar concentration of FSh and SEh in the half-cup EBN showed a significant difference in soluble protein concentration (*p* < 0.05), except for at 10,000 ppm, where the soluble protein concentration of SEh (189.80 mg/mL) was comparable to the protein concentration of FSh (179.80 mg/mL). This may be due to the presence of a strong peptide bond in the protein solution of EBNs, whereby any further increase in the concentration of EBN hydrolysate (10,000 ppm) was not able to increase the solubility of the protein due to the saturated state.

Enzymatic hydrolysis is often used to improve the solubility of the protein, and the protein degradation results in the formation of amino acids and peptides with smaller molecular masses easily absorbed by the digestive system. However, strong peptide–peptide interactions and the presence of glycoprotein that cannot be hydrolyzed result in less than 100% protein solubility [38]. Pepsin is a proteolytic enzyme secreted by the stomach, while pancreatin is produced by the pancreas, which contains other enzymes such as elastase, trypsin, and chymotrypsin proteases. Optimized extraction followed by simulated gastrointestinal digestion (pepsin and pancreatin hydrolysis) mimics the natural surroundings of the human digestive tract. This process could potentially increase the bioavailability of EBN bioactive peptides and subsequently absorb them through the gut to exert their functional effects.

The EBN hydrolysis process involves cleaving the peptide bonds within EBN proteins into bioactive glycopeptides using enzymes, thus improving the solubility of protein [4]. FS and SE extraction methods, in combination with enzymatic hydrolysis, increased the solubility of EBN extracts and hydrolysate in the respective samples. It is important for high protein food to be able to be well digested, metabolized, and absorbed by the human body, especially in athletes or patients with injuries or burns. Therefore, EBNs are a great alternative for substituting high protein sources into normal dietary intake.

### 3.6. Molecular Weight Distribution of Protein in Extracts and Hydrolysates of EBNs

**Figure 5:**
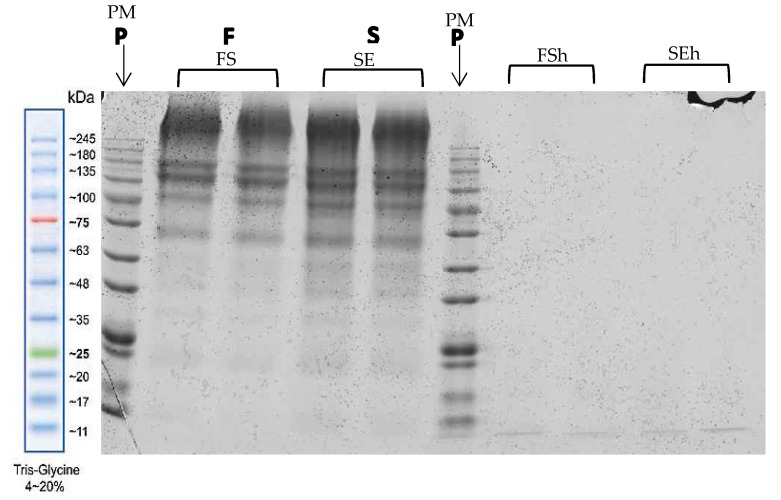
The molecular weight of protein for half-cup EBN extracts and hydrolysates. PM: Protein Marker; FS: Full stew; SE: Stew extract; FSh: Full stew hydrolysate; SEh: Stew hydrolysate.

Characterization of the protein in EBN extracts (FS and SE) and hydrolysates (FSh and SEh) were done by SDS-PAGE with Coomassie blue staining, as shown in Figure 5. The FS and SE extracts depicted a dominant band between 70 kDa and 180 kDa at the top of the gel and a smeared electrophoresis pattern down to 20 kDa, suggesting larger protein sizes had difficulty moving through the gel matrix. This is supported by [21], where the EBN protein band was clearly shown at the top of the SDS-PAGE gel matrix. These band patterns show that EBN extract contains large and complex molecular weight proteins. The protein molecular weight ranges from 100 to 135 kDa, indicating the presence of sialic acid, which has been reported as bound to protein with molecular weights of 106 and 128 kDa in EBN samples, known as ‘sialo-glycoprotein’ [18].

The FSh and SEh band shows that almost all protein is present in smaller peptides with a molecular weight of less than 11 kDa. Enzymatic hydrolysis breaks down the extracted protein of EBNs by uncoiling the protein and peptide bond, thus producing small molecules with lower molecular weight, as observe using SDS-PAGE. This is also due to the 7.0 KDa cut-off molecular weight of the dialysis tubing used to separate the mixture. The protein size of EBN extract was 140.8, 64.8, and 21.1 kDa, whereas the molecular weight of EBN glycoprotein was 140.8 and 64.8 kDa [39]. Raw EBNs depict more and distinct bands as compared to processed EBNs, where the EBN processing may have reduced the original amount of intact protein, thus resulting in different protein profiles [4]. The different types of EBN depict different protein profiles in SDS-PAGE, with 37–52 kDa as the most abundant protein size found in EBNs from Malaysia [5,36].

### 3.7. Protein and Peptide Profile of EBN

The protein and peptide sequences identified in FS and SE extracts from half-cup EBNs using LC-MS/MS are shown in Table 3. There were seven parent proteins identified in EBNs, namely 78 kDa glucose-regulated protein, lysyl oxidase-3, mucin-5AC-like, acidic mammalian chitinase-like (AMCase-like), 45 kDa calcium-binding protein, nucleobindin-2, and ovoinhibitor-like, with a molecular weight ranging from 39.15 to 181.68 kDa.

About 29 peptide sequences were found matched to the seven identified parent proteins, which had between 8 and 34 amino acid sequences in length and a molecular weight between 318.87 and 1054.19 Da. In this study, 78 kDa glucose-regulated protein was identified in half-cup EBN extract by LC-MS/MS, which has not been reported elsewhere. Meanwhile, six similar parent proteins were reported by [21], with the best-scored proteins being AMCase-like, mucin-5AC-like, and ovoinhibitor-like proteins. These similar findings may be attributed to the same double-boiled method of water extraction used in the present study.

The most abundant proteins identified in both FS and SE extract were Lysyl oxidase-3 and Mucin-5AC-like protein, which were also frequently detected in EBN samples [18,37]. The authenticity of EBNs from Malaysia, Indonesia, and Vietnam was achieved and was comparable with genuine EBNs, while the outcome of adulterated EBNs (being mixed with faked EBNs) was the opposite [40]. This authenticity assessment concluded that Muc-5AC protein data could serve as an internal marker of EBN fingerprinting with the highest discriminative power, due to this protein abundance in EBNs. However, there were peptide sequences that were only present in either FS or SE half-cup EBNs, corresponding to their parent protein. This might contribute to the different extraction methods used in digestion, resulting in different bioactive peptides.

The 78 kDa glucose-regulated protein or heat shock protein was found only in this study. By referring to their chaperone role, 78 kDa glucose-regulated protein was found to be responsible for regulating cell survival and apoptosis by degradation of misfolded protein and regulation of apoptotic activity, which significantly increases the neuroprotective effects in 6-OHDA-treated neuroblastoma cell model SH-SY5Y [10]. Lysyl oxidase-3 is an enzyme that is essential for the stabilization of collagen fibrils and elasticity of elastin, supporting the evidence on skin complexion improvement in EBN-supplemented ovariectomized rats [9].

Mucin-5Ac-like protein, with a molecular weight of 181.68 kDa, is a gel-forming glycoprotein produced by epithelial tissues and is a key component in most gel-like secretion, like saliva. Muc5Ac is secreted in the gastric and respiratory tract and functions to protect the mucosa from infection and chemical damage [21]. The AMCase-like protein could be highly expressed in the salivary gland of swiftlets and secreted into the saliva for digestion of chitin-containing insects or fungi [10]. The expression of chitinase in the human body could be triggered in response to allergies, possibly accounting for the anti-inflammatory properties of EBNs [11].

The 45 kDa calcium-binding protein consists of 361 amino acids and is associated with cellular and extracellular functions, from calcium homeostasis to calcium signaling pathways [41]. This protein is present in the brain regions and is highly expressed in astrocytes, neurons, oligodendrocyte progenitor cells, myelinating oligodendrocyte, microglia/macrophage, and endothelial cells; it might have fundamental functions in a cell’s calcium homeostasis [42].

Nucleobindin-2 is a calcium-binding protein with a suggested role in calcium level maintenance, eating regulation in the hypothalamus, and release of tumor necrosis factor from vascular endothelial cells. In another study on nueclobindinn-2, it was found able to control insulin sensitivity in the condition of caloric excess in the body; loss of this protein increased inflammation and insulin resistance upon high-fat feeding [43]. The protein ovoinhibitor is a serine protease inhibitor that can be found in egg whites. It plays a significant role in the antibacterial defense against *Bacillus subtilis* and *S. aureus* [44].

Proteomic analysis was influenced by the extraction method in projecting the biodiversity of the peptide profile. The application of different extraction methods for similar types of EBN resulted in a homogenous protein profile with respective matched peptide sequences. This input could be used as a future reference by researchers to explore the bioactive peptide and functional activities of EBNs. It is believed that there are still many unknown proteins, as limited databases are available for EBN proteins. Through the protein identification results, hidden potential bioactive peptides can be explored for nutritional and medicinal usage.

## 4. Conclusions

Numerous studies have proven the health benefits of EBN consumption, leading to an increase in the EBN farming business. However, the physicochemical and chemical characteristics of different shapes of EBNs have scarcely been reported. The findings from this study conclude that two different shapes (half-cup and stripe-shaped) of EBN comprise different physicochemical (physical, morphology, elemental composition, and microbial content) and chemical (crude protein content and amino acid profile) characteristics. In addition, optimum hydrolysis produces desirable effects on the extraction yield, soluble protein, sialic acid content, molecular weight, and protein profile. The extraction temperature (73 to 80 °C) applied in this study influences the yield, protein concentration, and solubility of EBNs. This contributes to the identification of potentially bioactive compounds, proteins, and peptides after different extraction methods. Thus, it is important to fully characterize EBNs before grading. Simultaneously, these findings can be a reference method for optimizing food preparation for EBN products by both industry and consumers. This research could be a reference for further studies on EBN biological activity.

## Figures and Tables

**Table 1:** Physical measurement, elemental distribution, and microbial content of half-cup and stripe-shaped EBN.

		Edible Bird Nest			
Parameter		Half-Cup		Stripe-Shaped	
		Macroscopic			
Measurement	*n* = 10	Range	Mean ± SD	Range	Mean ± SD
Height	cm	3.0–5.1	4.08 ± 0.72 ^a^	1.0–1.5	1.28 ± 0.15 ^b^
Length	cm	5.5–9.0	7.06 ± 1.33 ^a^	2.5–4.0	3.26 ± 0.48 ^b^
Weight	g	5.0–5.5	5.19 ± 0.18 ^a^	2.0–2.5	2.26 ± 0.18 ^b^
Element	*n* = 3	Microscopic Weight			
Carbon	%	4.78 ± 11.72 ^b^		28.49 ± 22.26 ^a^	
Oxygen	%	5.61 ± 13.75 ^b^		27.13 ± 21.30 ^a^	
Magnesium	%	ND		0.22 ± 0.53	
Calcium	%	72.94 ± 43.58 ^a^		44.16 ± 43.31 ^b^	
	Unit	Microbial Content			
Aerobic Plate Count	CFU/g	1.8 × 10^8^		1.8 × 10^8^	
Coliforms	CFU/g	1.4 × 10^4^		1.4 × 10^4^	
*Escherichia coli*	CFU/g	ND (<10)		ND (<10)	
*Salmonella*	in 25 g	Absent		Absent	
*Staphylococcus aureus*	MPN/g	ND (<3)		ND (<3)	
Yeasts and molds	CFU/g	67		ND (<10)	

Data shown are means ± standard deviation. ^a b^ Different superscript letters in the same row indicate a statistically significant difference between half-cup and striped-shaped EBNs (*p* < 0.05). ND: Not detected; <: Less than the minimum detection limit reported; CFU: Colony-forming unit; MPN: Most probable number.

**Table 2:** Crude protein content and amino acid profile of EBNs.

Parameter		This Study		[2]	[19]	
		Half-Cup	Stripe-Shaped			
Location	Compound (%)	Terengganu		Pahang	Perak, Penang, Kedah	Sabah, Sarawak
Crude protein		56.96 ± 0.09 ^a^	54.70 ± 0.16 ^b^	58.55 ± 0.62	53.8 ± 0.18	52.8 ± 1.04
	Arginine	6.74	6.61	3.80	4.50	4.10
Essential amino acids (EAAs)	Histidine	4.61	4.66	1.40	1.50	1.40
	Isoleucine	3.38	3.38	3.40	0.60	0.50
	Leucine	6.74	6.80	5.30	2.70	2.50
	Lysine	4.49	4.34	5.40	1.30	1.10
	Methionine	2.85	2.90	2.20	0.70	0.70
	Phenylalanine	6.23	6.33	2.70	2.30	2.20
	Threonine	6.73	6.66	2.90	2.70	2.40
	Valine	7.58	7.43	3.30	1.60	1.40
	Total EEA	49.35	49.11	30.40	17.90	16.30
	Alanine	4.36	4.43	3.90	1.30	1.20
Non-essential amino acids (nEAAs)	Aspartic acid	9.14	8.94	6.30	4.00	3.70
	Cysteine	1.37	1.29	1.70	1.10	1.40
	Glutamic acid	11.20	11.68	9.60	3.00	2.60
	Glycine	3.68	3.66	2.50	1.50	1.60
	Proline	6.95	6.84	2.90	3.20	2.90
	Serine	8.60	8.37	2.40	4.30	4.00
	Tyrosine	5.34	5.64	2.90	2.70	2.60
	Total nEAA	50.64	50.85	32.20	21.10	20.00

Values are expressed as mean (%) ± S.D. Means with different superscript letters in a row indicate a significant difference between half-cup and striped-shaped EBNs (*p* < 0.05).

**Table 3:** Protein and peptide sequences identified by LC-MS/MS in the full stew (FS) and stew extracts (SE) of half-cup EBNs.

Protein	MW (kDa)	Parent Protein, (Accession No.) ^a^	Score ^b^		Sample	Peptide Sequence ^c^	Position ^d^	mz^−1^	Biological Function (Annotated in UniProt/SwissProt)
			Full Stew(FS)	Stew (SE)					
1	67.72	78 kDa glucose- regulated protein(A0A0A0B169)	204.84	39.01	FS/SE	[K]. SQIFSTASDNQPTVTIK. [V]	407–423	918.96	Stress response
2	53.97	Lysyl oxidase homolog 3 (A0A0A0B371)	538.63	538.63	FS/SE	[R]. QLPVTEGIVEVR. [Y]	97–108	670.38	Stabilization of collagen fibrils, elasticity of mature elastin
					FS/SE	[R]. IPGFKDSNVIETEQSHVEEVR. [L]	66–86	604.05	
					FS/SE	[R]. LRPVVSGAR. [R]	87–95	318.87	
					FS/SE	[K]. DSNVIETEQSHVEEVR. [L]	71–86	935.94	
					**SE**	**RQLPVTEGIVEVR. [Y]**	**96–108**	**499.29**	
3	181.68	Mucin-5AC (R7VT28)	668.96	326.51	FS/SE	[K]. GVLLTGWR. [S]	700–707	451.27	Gel-forming glycoprotein of gastric and respiratory tract epithelia
					FS/SE	[K]. TTSGVIEGTSAAFGNTWK. [T]	598–615	913.95	
					FS/SE	[K]. SPYEDFNIQIR. [R]	115–125	691.34	
					FS/SE	[R]. SQSVVGNVLEFANSWK. [V]	1064–1079	882.95	
					**FS**	**[R]. GSVLLDGK. [L]**	**152–159**	**394.72**	
4	42.04	Acidic mammalian chitinase-like (A0A0A0APZ4	198.81	213.39	FS/SE	[K]. LLVGFPTYGR. [N]	243–252	561.82	Chitin degradation, inflammatory response against pathogen
					FS/SE	[K]. FSTMVSTPQNR. [Q]	94–104	634.31	
					FS/SE	[K]. YPLITTLK. [N]	360–367	474.79	
5	39.15	45 kDa calcium- binding protein (A0A0A0AQY4)	119.81	147.78	FS/SE	[K]. NNEELKIDEETQEVLDNLKDR. [W]	153–173	636.82	Exocytosis
					FS/SE	[K]. LTLSEFISLPVGTVENQQAQDIDDDWVK. [D]	223–250	1054.19	
					FS/SE	[K]. TDEHFQEAVEENK. [M]	101–113	525.9	
					FS/SE	[K]. EMEEFEEDSEPR. [K]	56–67	763.8	
					FS/SE	[R]. AVDPDGDGHVSWDEYK. [I]	118–133	895.39	
					**FS**	**[K]. QMIAVADENQNHHLELEEILK. [Y]**	**291–311**	**619.31**	
					**SE**	**[K]. IKNNEELKIDEETQEVLDNLK. [D]**	**151–171**	**629.33**	
6	53.47	Nucleobindin-2 (A0A0A0AZD6)	38.59	73.77	FS/SE	[K]. EVWEEADGLDPNEFDPK. [T]	231–247	995.44	Calcium homeostasis
					FS/SE	[R]. LVTLEEFLR. [A]	312–320	560.32	
					FS/SE	[K]. AATSDLENYDK. [T]	161–171	613.78	
					**FS**	**[K]. LHDVNNDGFLDEQELEALFTK. [E]**	**252–272**	**816.39**	
					**SE**	**[K]. VENPDTGLYYDEYLR. [Q]**	**45–59**	**923.93**	
					**SE**	**[K]. QFEHLNHQNPDTFEPK. [D]**	**138–153**	**495.99**	
					**SE**	**[K]. LQTADIEEIK. [S]**	**75–84**	**580.31**	
7	43.22	Ovoinhibitor (A0A099ZXZ8)	46.77	41.4	FS/SE	[R]. QLMACTMIYDPVCGTDGVTYASECTLCAHNLEHR. [T]	344–377	998.43	Anti-viral

MW: Molecular weight; ^a^ Accession number is a reference to *Charadrius vociferus* in SwissProt database; ^b^ Protein score contributed by each peptide matched in SwissProt database; ^c^ Peptide sequences were matched with *C. vociferous* protein databases in SwissProt; ^d^ Position of the peptide inside the parent protein; Peptide sequences with **BOLD** text only identified in respective extract.

## Data Availability

The physicochemical analysis (physical, morphology, elemental composition, and microbial content), chemical analysis (crude protein and amino acid), sialic acid analysis and extraction effects on soluble protein and molecular weight data used to support the findings are provided within the article.
